# Experiences of Interpersonal Violence in Sport and Perceived Coaching Style Among College Athletes

**DOI:** 10.1001/jamanetworkopen.2023.50248

**Published:** 2024-01-16

**Authors:** Cheryl K. Zogg, Edward B. Runquist, Michael Amick, Gabrielle Gilmer, Jeffery J. Milroy, David L. Wyrick, Katharina Grimm, Yetsa A. Tuakli-Wosornu

**Affiliations:** 1 Department of Surgery, Yale School of Medicine, New Haven, Connecticut; 2 Department of Sports Medicine, Drexel School of Medicine, Philadelphia, Pennsylvania; 3 Department of Orthopaedics and Rehabilitation, Yale School of Medicine, New Haven, Connecticut; 4 Cellular and Molecular Pathology Graduate Program, Department of Pathology, University of Pittsburgh School of Medicine, Pittsburgh, Pennsylvania; 5 Institute to Promote Athlete Health & Wellness, University of North Carolina Greensboro, Greensboro; 6 Sports Equity Lab, New Haven, Connecticut; 7 Department of Social and Behavioral Sciences, Yale School of Public Health, New Haven, Connecticut

## Abstract

**Importance:**

Concern about interpersonal violence (IV) in sport is increasing, yet its implications remain poorly understood, particularly among currently competing college athletes.

**Objective:**

To document the self-reported prevalence of IV in college sports; identify associated risk factors; examine potential consequences associated with athletes’ psychosocial well-being, emotional connection to their sport, and willingness to seek help; and explore the associations between IV reporting and perceived variations in coaching styles.

**Design, Setting, and Participants:**

This survey study analyzes results of the 2021 to 2022 National Collegiate Athletic Association (NCAA) myPlaybook survey, which was administered from July to December 2021 to 123 colleges and universities across the US. Participants were NCAA athletes aged 18 to 25 years who were current players on an NCAA-sanctioned team.

**Exposures:**

Self-reported demographic characteristics (eg, athlete gender identity and sexual orientation) and perceived differences in supportive vs abusive coaching styles (eg, athlete autonomy, team culture, and extent of abusive supervision).

**Main Outcomes and Measures:**

The primary outcome was self-reported experiences of IV in sport during the college sports career of currently competing college athletes. Types of IV considered were physical abuse, financial abuse, sexual abuse, psychological or emotional abuse, and neglect or abandonment. Outcomes potentially affected by IV were assessed with 4 questionnaires.

**Results:**

A total of 4119 athletes (mean [SD] age, 19.3 [1.5] years; 2302 males [55.9%]) completed the survey (response rate, 21.2%). One in 10 athletes (404 of 4119 [9.8%]) reported experiencing at least 1 type of IV during their college sports career, of whom two-thirds (267 [6.5%]) experienced IV within the past 6 weeks. On multivariable analysis, female gender identity (odds ratio [OR], 2.14; 95% CI, 1.46-3.13), nonheterosexual sexual orientation (OR, 1.56; 95% CI, 1.01-2.42), increasing age beyond 18 years (OR, 1.13; 95% CI, 1.01-1.30), increasing year of NCAA eligibility beyond the first year (OR, 1.19; 95% CI, 1.02-1.39), and participation in select sports (eg, volleyball: OR, 2.77 [95% CI, 1.34-5.72]; ice hockey: OR, 2.86 [95% CI, 1.17-6.95]) were independently associated with IV. When exposed to IV, college athletes reported experiencing consistently worse psychosocial outcomes, including increased burnout (mean difference on a 5-point Likert scale, 0.75; 95% CI, 0.63-0.86; *P* < .001) and an expressed desire to consider quitting their sport (mean difference, 0.81; 95% CI, 0.70-0.92; *P* < .001). They were not, however, less willing to seek help. Differences in coaching style were associated with differences in IV reporting. In risk-adjusted linear regression models, having a more supportive coach was associated with a 7.4 (95% CI, 6.4-8.4) absolute percentage point decrease in athletes’ probability of reporting experiencing IV. In contrast, having a more abusive coach was associated with up to a 15.4 (95% CI, 13.8-17.1) absolute percentage point increase in athletes’ probability of reporting experiencing IV.

**Conclusions and Relevance:**

Results of this survey study suggest that IV is associated with marked changes in the psychosocial health and emotional well-being of college athletes, particularly those who identify as female and with nonheterosexual sexual orientations. Variations in coaching style have the potential to alter these associations. Ongoing efforts are needed to leverage the unique position that coaches hold to help reduce IV and create safe places where all college athletes can thrive.

## Key Points

**Question:**

What is the prevalence of US college athletes’ self-reported experiences of interpersonal violence (IV) during their college sports career, and how does the likelihood of experiencing IV vary with perceived differences in coaching style?

**Findings:**

In this survey study involving 4119 currently competing US college athletes, 1 in 10 athletes reported experiencing IV during their college sports career. Differences in coaching style were associated with differences in IV incidence, serving as both a risk factor for (eg, more abusive coaching) and protector against (eg, more supportive coaching) experiencing IV.

**Meaning:**

Findings of this study suggest that IV is associated with marked changes in the psychosocial health and emotional well-being of athletes; ongoing efforts are needed to leverage the unique position that coaches hold to help reduce IV and create safe places where all college athletes can thrive.

## Teaser

This survey study examines the prevalence of and factors associated with self-reporting of interpersonal violence in US collegiate sports among current student athletes.

## Introduction

Sport participation has long been recognized as a major factor in the psychosocial health and emotional well-being of athletes.^1^ However, in recent years, experiences of sports-related abuse, formally termed *interpersonal violence* (IV),^2^ have begun to garner increasing attention. Particular concern is centered on the increasing number of athlete-reported instances of harmful interactions between student athletes and coaches, a relationship that can be among the most meaningful and influential in a young athlete’s life.^3,4^ Defined by the World Health Organization as “the intentional use of physical force or power, threatened or actual,” against another person “that either results in or has a high likelihood of resulting in injury, death, psychological harm, maldevelopment, or deprivation,”^2^ IV is a broadly inclusive, multifaceted term. The term rose to prominence in both the developmental health and sports medicine literature as a means to describe the range of abusive behaviors experienced by athletes across a spectrum of distinct yet interrelated domains, including physical abuse, financial abuse, sexual abuse, psychological or emotional abuse, and neglect or abandonment.^5^

Recognizing the problem that IV could pose, leading international governing bodies in sport have started to formally acknowledge the harm that IV can have on the lives of affected athletes.^1,6^ Among elite athletes competing at the top of their sport, research suggests that as many as 75% will experience IV at some point during their career,^7^ particularly those who identify as being female,^7,8^ having a disability,^9^ belonging to historically underrepresented racial and ethnic groups,^10,11^ and having a nonheterosexual sexual orientation.^9^ Among youth or adolescent^11,12,13,14,15^ and collegiate^16,17,18^ athletes, the prevalence of IV has been reported to be upward of 33%, with a substantial proportion of the literature focusing on experiences of sexual abuse as a form of IV.^16,17,18,19,20^

The extent to which exposure to IV is associated with athletes’ psychosocial or emotional health and performance-related outcomes is less clear, particularly among US college athletes who are currently competing. Literature suggests that among college students in general, experiences of IV are often associated with higher prevalence of anxiety, depression, and posttraumatic stress disorder^21,22^; worse academic performance^23,24^; greater extents of suicidal ideation^25,26^; and overall low rates of mental health service use.^27,28,29^ Among athletes, IV has been associated with numerous adverse outcomes, including higher reported rates of distress, disassociation, and challenges with interpersonal relationships; greater rates of sports dropout; low self-esteem; and a higher prevalence of psychiatric diagnoses (eg, posttraumatic stress disorder and eating disorders).^30,31,32,33,34,35^ Tasked with the added challenge of balancing athletic, academic, financial, and scholarship obligations,^36^ currently competing US college athletes are unique. They have responsibilities that are distinct from the responsibilities of many of their similarly aged recreational sport–playing, college-attending, and professional peers while remaining as 18- to 25-year-old college students at a developmentally critical period of their lives.^37,38,39^ The implications of IV for such a population in such a high-intensity sports setting are not completely understood.

Given the need to better understand the risk factors and potential consequences associated with experiencing IV among college athletes, we partnered with the National Collegiate Athletic Association (NCAA) to (1) document the self-reported prevalence of IV in sport among currently competing college athletes; (2) identify associated (independent) risk factors; (3) examine potential consequences related to athletes’ psychosocial well-being, emotional connection to their sport, and willingness to seek help; and (4) explore the associations between IV reporting and perceived variations in (supportive vs abusive) coaching styles. As a secondary objective, we examined how reported experiences of IV varied among athletes who identified as belonging to groups historically underrepresented in US colleges and sports on the basis of their race and ethnicity, gender identity, sexual orientation, or disability status.

## Methods

### Data Source and Study Population

Data used in this study were collected as a part of the 2021 to 2022 NCAA myPlaybook survey, an anonymous electronic survey annually administered to all NCAA athletes at participating schools.^40,41^ In 2021 to 2022, the survey included 123 colleges and universities across the US.^40^ The survey was administered by the University of North Carolina Greensboro Institute to Promote Athlete Health & Wellness from July to December 2021 using the online survey software Qualtrics. To participate, athletes were required to be aged 18 to 25 years at the time of survey completion and a current member of an NCAA-sanctioned sport or athletic team. Completion of the survey required participants to provide written informed consent. The Institutional Review Board of the University of North Carolina Greensboro approved this study. We followed the American Association for Public Opinion Research (AAPOR) reporting guideline.

Survey questions about IV were added to myPlaybook in 2021 (eAppendix in Supplement 1). Previously published questionnaires on IV in sport^16,42,43,44^ were used to inform the development of a succinct, novel 5-item screening questionnaire. Intended to capture instances of self-reported IV among currently competing college athletes in a nonintrusive and nonstigmatizing manner, the questionnaire asked about personal experiences of IV across 5 recognized types: physical abuse, financial abuse, sexual abuse, psychological or emotional abuse, and neglect or abandonment.^16,42,43,44^ Operational definitions for these 5 IV types are provided in the eAppendix in Supplement 1.

The questionnaire was designed as a screening-style instrument with intentionally broad, nontriggering questions instead of in-depth scenario-based questions. This design was chosen to comply with current survey guidelines for young athletes created by members of the sports and exercise medicine community committed to focus on IV screening that is “athlete-centered, trauma-informed, human-rights- and ethics-based, accountable to the complexities of sport, and [able to] balance the potential benefits of screening, study recruitment, and [attainment of] population-level prevalence data against the ethical obligation to provide safety-net environments and therapeutic resources once interpersonal violence is identified”^45^^(p1)^ in instances wherein survey completion might otherwise be triggering to individuals in the middle of experiencing IV.^45,46^

### Variable Definitions

The primary outcome was self-reported experiences of IV in sport during currently competing college athletes’ college sports career. Secondary outcomes were reported experiences of IV during the past 6 weeks, experiences of each type of IV, and experiences of multiple types of IV.

Differences in self-reported demographic characteristics were used to identify crude (unadjusted) and multivariable (risk-adjusted) associations with IV. Considered demographic characteristics included self-reported variations in athlete race and ethnicity, gender identity, sexual orientation, disability status, international student status, age, year of NCAA eligibility (year in school), NCAA division, and NCAA-sanctioned sport. Coach race and ethnicity and coach gender identity were also considered. Investigator-defined categories for each are listed in the eAppendix in Supplement 1.

Outcomes potentially affected by IV were assessed using 4 questionnaires included as part of myPlaybook.^40,41^ These questionnaires examined differences in athletes’ perceived athletic performance, team cohesion, emotional connection to their sport, and reported willingness to seek help. Perceived athletic performance^47,48^ was evaluated with the 10-point Athlete’s Subjective Performance Scale, with higher values indicating better subjective performance. Team cohesion^49^ was evaluated with the 9-point Group Environment Questionnaire scale, with higher values indicating greater group inclusion. Emotional connection to their sport^50^ was evaluated with the 5-point SafeSport scale, with higher values indicating less emotional connection. Willingness to seek help^51^ was evaluated with the 5-point Self-Stigma of Seeking Help scale, with higher values indicating lower willingness to seek help. Differences in coaching styles were evaluated using 3 questionnaires: Coach Autonomy Supporting Behaviors Scale, a 7-point scale assessing support of athlete autonomy, with higher scores indicating greater autonomy; InSideOut Initiative: Team Culture, a 5-point scale assessing team culture, with higher scores indicating more supportive team culture; and Abusive Supervision Scale, a 5-point scale assessing the extent of abusive supervision, with higher scores indicating more abusive supervision.^40,41^

### Statistical Analysis

Reported experiences of IV and each of its 5 types were summarized using frequencies and percentages for categorical variables. Stratified differences were compared using 2-sided χ^2^ tests (Fischer exact tests in cell counts fewer than 5). Differences in demographic characteristics were similarly compared. Risk-adjusted differences were assessed using multivariable logistic regression (outcome: IV; explanatory variables: included demographic characteristics). Associations between IV and questionnaire-derived outcomes were assessed using multivariable linear regression (outcome: outcome in question; explanatory variable: IV; confounders: considered demographic characteristics). Relative contributions to each outcome from each of the 5 types of IV were calculated using structural equation models. Differences in athletes’ probability of reporting IV based on differences in perceived coaching styles were summarized using multivariable linear regression (outcome: probability of experiencing IV [0%-100%]; explanatory variable: perceived coaching style; confounders: considered demographic variables).

Data cleaning and statistical analyses were conducted using Stata, version 17.0 (StataCorp LLC). Two-sided *P* < .05 with Bonferroni corrections (α = .05/number of comparisons) were considered to be significant.

## Results

A total of 19 527 currently competing college athletes were invited to participate in the survey. Of these athletes, 4337 started the survey (initial response rate, 22.1%) and 4119 provided complete information on the variables needed to conduct the analysis (completed survey response rate, 21.2%). Athletes who completed the survey (n = 4119) had a mean (SD) age of 19.3 (1.5) years; of these athletes, 1795 self-identified as female (43.6%), 2302 as male (55.9%), and 19 (0.5%) as transgender (n = 3), nonbinary (n = 9), or unspecified other gender identities (n = 7) (Table 1). Due to the limited number of responses, athletes who identified with other gender identities were excluded from the binary categorization of male vs female athletes used in multivariable and stratified analyses. One hundred four athletes (2.5%) declined to disclose their sexual orientation; they were excluded from the binary categorization of heterosexual vs nonheterosexual athletes used in multivariable and stratified analyses.

**Table 1.** Overall Demographic Characteristics and Unadjusted Associations Between Demographic Factors and Self-Reported Experiences of Interpersonal Violence (IV)

Characteristic	No. (%)			*P* value^a^
	Overall (n = 4119)	No IV (n = 3715)	Any form of IV (n = 404)	
Athlete age, y				
18	1712 (41.6)	1606 (43.2)	107 (26.4)	<.001^b^
19	978 (23.7)	887 (23.9)	91 (22.5)	
20	491 (11.9)	422 (11.4)	69 (17.0)	
21	484 (11.8)	401 (10.8)	83 (20.6)	
22	284 (6.9)	245 (6.6)	39 (9.7)	
≥23	170 (4.1)	154 (4.1)	16 (3.9)	
Mean (SD) age, y	19.3 (1.5)	19.3 (1.5)	19.8 (1.5)	<.001^b^
Athlete race and ethnicity^c^				
American Indian or Alaskan Native	43 (1.0)	43 (1.2)	0	.24
Asian	65 (1.6)	56 (1.5)	8 (2.1)	
Hispanic or Latino	313 (7.6)	285 (7.7)	28 (7.1)	
Native Hawaiian or Pacific Islander	19 (0.4)	17 (0.5)	1 (0.3)	
Non-Hispanic Black	764 (18.5)	695 (18.7)	69 (17.0)	
Non-Hispanic White	2662 (64.6)	2396 (64.5)	266 (65.8)	
Other^b^	30 (0.7)	27 (0.7)	2 (0.5)	
Multiracial	224 (5.4)	194 (5.2)	30 (7.3)	
Athlete gender identity				
Male	2302 (55.9)	2143 (57.7)	158 (39.2)	<.001^b^
Female	1795 (43.6)	1554 (41.8)	241 (60.3)	
Transgender	3 (0.1)	3 (0.1)	0	
Nonbinary	9 (0.1)	9 (0.1)	0	
Other^d^	7 (0.3)	6 (0.3)	1 (0.5)	
Athlete sexual orientation^e^				
Straight or heterosexual	3775 (94.0)	3428 (94.7)	347 (88.2)	<.001^b^
Gay, lesbian, or homosexual	83 (2.1)	73 (2.0)	10 (2.4)	
Bisexual or pansexual	123 (3.1)	98 (2.7)	25 (6.5)	
Asexual	12 (0.3)	11 (0.3)	1 (0.3)	
Questioning	22 (0.6)	12 (0.3)	11 (2.7)	
Athlete with disability	106 (2.6)	95 (2.3)	11 (5.0)	.001^b^
Coach race and ethnicity^c^				
American Indian or Alaskan Native	2 (0.1)	2 (0.1)	0	.08
Asian	25 (0.6)	20 (0.6)	4 (1.1)	
Hispanic or Latino	64 (1.6)	56 (1.5)	7 (1.8)	
Native Hawaiian or Pacific Islander	11 (0.3)	6 (0.2)	4 (1.1)	
Non-Hispanic Black	496 (12.0)	442 (11.9)	54 (13.4)	
Non-Hispanic White	3289 (79.9)	2976 (80.1)	313 (77.4)	
Other^d^	17 (0.4)	15 (0.4)	2 (0.5)	
Multiracial	23 (0.6)	22 (0.6)	1 (0.3)	
Uncertain or multiple	192 (4.7)	174 (4.7)	18 (4.5)	
Coach gender identity				
Male	3244 (78.8)	2952 (79.5)	292 (72.4)	.006
Female	848 (20.6)	740 (19.9)	108 (26.8)	
Uncertain or multiple	26 (0.6)	23 (0.6)	3 (0.8)	
International student status	502 (12.2)	451 (12.1)	51 (12.6)	.81
NCAA division				
Division I	1078 (26.2)	971 (26.1)	107 (26.5)	.30
Division II	1986 (48.2)	1804 (48.6)	182 (44.9)	
Division III	1055 (25.6)	940 (25.3)	115 (28.6)	
Year of NCAA eligibility or year in school				
1st	2667 (64.8)	2479 (66.7)	188 (46.5)	<.001^b^
2nd	538 (13.0)	471 (12.7)	66 (16.5)	
3rd	439 (10.7)	366 (9.8)	74 (18.3)	
4th	304 (7.4)	248 (6.7)	56 (13.8)	
5th	171 (4.2)	151 (4.1)	20 (5.0)	
Mean (SD) year of eligibility	1.7 (1.2)	1.7 (1.1)	2.1 (1.3)	<.001^b^
Athlete sport				
Baseball	433 (10.5)	416 (11.2)	17 (4.2)	<.001^b^
Basketball	380 (9.2)	353 (9.5)	27 (6.6)	
Beach volleyball	18 (0.4)	14 (0.4)	4 (1.1)	
Bowling	15 (0.4)	12 (0.3)	3 (0.8)	
Cheer	22 (0.5)	17 (0.5)	4 (1.1)	
Cross country	160 (3.9)	151 (4.1)	9 (2.1)	
Fencing	10 (0.2)	9 (0.2)	1 (0.3)	
Field hockey	57 (1.4)	50 (1.4)	6 (1.6)	
American football	652 (15.8)	596 (16.0)	56 (14.0)	
Golf	126 (3.1)	116 (3.1)	10 (2.4)	
Ice hockey	86 (2.1)	74 (2.0)	12 (2.9)	
Lacrosse	324 (7.9)	295 (7.9)	30 (7.4)	
Rowing	26 (0.6)	24 (0.6)	2 (0.5)	
Skiing	26 (0.6)	24 (0.6)	2 (0.5)	
Soccer (football)	510 (12.4)	451 (12.1)	59 (14.5)	
Softball	296 (7.2)	263 (7.1)	33 (8.2)	
Swimming and diving	209 (5.1)	178 (4.8)	31 (7.7)	
Tennis	127 (3.1)	104 (2.8)	22 (5.5)	
Track and field (indoor)	148 (3.6)	127 (3.4)	21 (5.3)	
Track and field (outdoor)	162 (3.9)	149 (4.0)	13 (3.2)	
Volleyball	180 (4.4)	149 (4.0)	31 (7.7)	
Water polo	15 (0.4)	14 (0.4)	1 (0.3)	
Wrestling	49 (1.2)	47 (1.3)	2 (0.5)	
Other or multiple sports	89 (2.2)	82 (2.2)	7 (1.9)	
Sport currently in season	2171 (52.7)	1947 (52.4)	224 (55.5)	.24

Abbreviation: NCAA, National Collegiate Athletic Association.

^a^ Two-sided *P* values calculated using χ^2^ tests (Fischer exact test in cell counts fewer than 5), Bonferroni-corrected threshold: 2-sided *P* < .005.

^b^ Statistically significant.

^c^ Race and ethnicity were self-reported in survey.

^d^ “Other” was selected by respondents when the provided options did not adequately reflect their identity.

^e^ Excludes 104 athletes who declined to disclose their sexual orientation.

### Prevalence of IV in Sports

Among 4119 athletes, 404 (9.8%) reported experiencing IV during their college sports career and 267 (6.5%) reported experiencing IV within the past 6 weeks (Table 2). The most prevalent types of IV were psychological or emotional abuse (256 [6.2%]), neglect or abandonment (240 [5.8%]), and financial abuse (115 [2.8%]). One in 100 athletes (53 [1.3%]) reported experiencing physical abuse. A similar number (35 [0.9%]) acknowledged sexual abuse. One in 20 athletes (191 [4.6%]) reported multiple types of abuse.

**Table 2.** Prevalence of Interpersonal Violence (IV), Stratified by Athlete Race and Ethnicity, Gender Identity, Sexual Orientation, and Disability Status

Factor	Overall, No. (%)	Prevalence of IV, %											
		Race and ethnicity			Gender identity^a^			Sexual orientation^b^			Disability status		
		White	Other^c^	*P* value^d^	Male	Female	*P* value^d^	Heterosexual	Nonheterosexual	*P* value^d^	Without disability	With disability	*P* value^d^
Total participants, No. (%)	4119	2663 (64.7)	1456 (35.3)		2299 (55.8)	1801 (43.7)		3780 (94.2)	235 (5.9)		4013 (97.4)	106 (2.6)	
**Any form of IV**													
During college sports career	404 (9.8)	10.2	9.7	.62	7.1	14.0	<.001^e^	9.3	17.4	<.001^e^	9.6	18.9	.001^e^
In the past 6 weeks	267 (6.5)	6.3	7.0	.36	4.8	8.8	<.001 ^e^	6.0	13.2	<.001^e^	6.1	19.8	<.001^e^
**Physical**													
During college sports career	53 (1.3)	0.9	1.7	.03	1.4	1.0	.28	1.2	1.1	.86	1.3	1.0	.77
In the past 6 weeks	34 (0.8)	0.5	1.1	.04	0.9	0.7	.500	0.8	0.0	.24	0.8	1.0	.88
**Financial**													
During college sports career	115 (2.8)	2.1	3.9	.002	2.9	2.7	.72	2.7	2.1	.64	2.7	5.8	.06
In the past 6 weeks	69 (1.7)	1.2	2.4	.009	1.9	1.4	.27	1.7	0.5	.23	1.6	4.9	.01
**Sexual**													
During college sports career	35 (0.9)	0.7	1.1	.19	0.7	0.9	.51	0.8	1.1	.63	0.8	1.9	.23
In the past 6 weeks	26 (0.6)	0.4	0.9	.03	0.7	0.4	.19	0.6	0.0	.31	0.6	1.0	.67
**Psychological or emotional**													
During college sports career	256 (6.2)	6.3	6.1	.83	3.5	9.7	<.001^e^	5.7	12.2	<.001^e^	5.9	16.5	<.001^e^
In the past 6 weeks	158 (3.8)	3.7	4.1	.49	2.4	5.5	<.001^e^	3.4	8.6	<.001^e^	3.5	16.5	<.001^e^
**Neglect or abandonment**													
During college sports career	240 (5.8)	6.3	5.2	.16	3.7	8.8	<.001^e^	5.5	10.1	.008	5.7	11.5	.01
In the past 6 weeks	155 (3.8)	3.6	4.1	.46	2.4	5.5	<.001^e^	3.5	7.5	.005	3.6	8.7	.008
**Multiple types**													
During college sports career	191 (4.6)	4.8	4.6	.75	2.9	7.2	<.001^e^	2.9	7.2	<.001^e^	4.4	13.2	<.001^e^
In the past 6 weeks	101 (2.5)	2.4	2.7	.46	1.7	3.6	<.001^e^	2.3	2.6	.79	2.3	6.6	.005

^a^ Excludes 19 athletes who identified as transgender, nonbinary, or unspecified other.

^b^ Excludes 104 athletes who declined to disclose their sexual orientation.

^c^ Other race and ethnicity included American Indian or Alaskan Native, Asian, Hispanic or Latino, Native Hawaiian or Pacific Islander, non-Hispanic Black, other, and multiracial.

^d^ Two-sided *P* values calculated using χ^2^ tests (Fischer exact test in cell counts fewer than 5), Bonferroni-corrected threshold: 2-sided *P* < .005.

^e^ Statistically significant.

Stratification based on self-reported differences in athlete identity revealed the following results. First, there were no significant differences on the basis of race and ethnicity. Second, female athletes were significantly more likely than male athletes to report both IV (14.0 vs 7.1%; *P* < .001) and multiple types of IV (7.2 vs 2.9%; *P* < .001). The differences were primarily associated with greater extents of psychological or emotional abuse (9.7 vs 3.5%; *P* < .001) and neglect or abandonment (8.8 vs 3.7%; *P* < .001). Third, nonheterosexual athletes were significantly more likely than heterosexual athletes to report IV (17.4 vs 9.3%; *P* < .001) and multiple types of IV (7.2 vs 2.9%; *P* < .001). The differences were primarily associated with greater extents of psychological or emotional abuse (12.2 vs 5.7%; *P* < .001). Fourth, athletes with disabilities were significantly more likely than athletes without disabilities to report IV (18.9 vs 9.6%; *P* = .001) and multiple types of IV (13.2 vs 4.4%; *P* < .001). The differences were primarily associated with greater extents of psychological or emotional abuse (16.5 vs 5.9%; *P* < .001).

### Demographic Factors and IV

Compared with athletes not reporting IV (n = 3715), athletes who reported IV (n = 404) were more likely to identify as female (60.3 vs 41.8%; *P* < .001) and have nonheterosexual sexual orientation (11.8 vs 5.3%; *P* < .001) (Table 1). Athletes who reported experiencing IV were also more likely to have disabilities (5.0 vs 2.3%; *P* = .001), be older (eg, >18 years vs 18 years: 73.6% vs 56.8%; *P* < .001), be further into their 5 years of NCAA eligibility (second to fifth year vs first year: 53.5% vs 33.3%; *P* < .001), and participate in sports involving full contact (eg, American football or ice hockey) or predominately female athletes (eg, cheer or volleyball).

On multivariable analysis (Table 3), the following demographic factors remained independently associated with IV: female gender identity (odds ratio [OR], 2.14; 95% CI, 1.46-3.13); nonheterosexual sexual orientation (OR, 1.56; 95% CI, 1.01-2.42); increasing age, with each year relative to age 18 years (OR, 1.13; 95% CI, 1.01-1.30); increasing year of NCAA eligibility, with each year relative to athletes’ first year (OR, 1.19; 95% CI, 1.02-1.39); and participation in select sports (eg, American football: OR, 2.91 [95% CI, 1.59-5.31]; ice hockey: OR, 2.86 [95% CI, 1.17-6.95]; volleyball: OR, 2.77 [95% CI, 1.34-5.72]).

**Table 3.** Multivariable Risk-Adjusted Associations Between Demographic Factors and Self-Reported Experiences of Interpersonal Violence

Factor	Risk-adjusted OR (95% CI)^a^
Athlete race and ethnicity	
White	1.00 [Reference]
Other^b^	0.92 (0.67-1.26)
Athlete gender identity	
Male	1.00 [Reference]
Female	2.14 (1.46-3.13)^c^
Athlete sexual orientation	
Straight or heterosexual	1.00 [Reference]
Nonheterosexual	1.56 (1.01-2.42)^c^
Athlete with disability	1.42 (0.75-2.71)
Coach race and ethnicity	
White	1.00 [Reference]
Other^b^	1.21 (0.89-1.67)
Coach gender identity	
Male	1.00 [Reference]
Female	1.14 (0.53-2.46)
International student status	0.81 (0.55-1.18)
NCAA division	
Division I	1.07 (0.76-1.50)
Division II	1.03 (0.77-1.39)
Division III	1.00 [Reference]
Additional year of age	1.13 (1.01-1.30)^c^
Additional year of NCAA eligibility	1.19 (1.02-1.39)^c^
Athlete sport	
Baseball	1.00 [Reference]
Basketball	1.27 (0.62-2.57)
Beach volleyball	3.64 (1.00-13.29)^c^
Bowling	3.34 (0.73-15.28)
Cheer	3.37 (0.93-12.28)
Cross country	0.93 (0.37-2.31)
Fencing	1.54 (0.16-14.59)
Field hockey	1.84 (0.62-5.42)
American football	2.91 (1.59-5.31)^c^
Golf	1.76 (0.73-4.25)
Ice hockey	2.86 (1.17-6.95)^c^
Lacrosse	1.69 (0.85-3.37)
Rowing	0.64 (0.08-5.23)
Skiing	1.23 (0.25-6.08)
Soccer (football)	2.32 (1.24-4.37)^c^
Softball	1.59 (0.79-3.23)
Swimming and diving	3.26 (1.63-6.51)^c^
Tennis	3.11 (1.42-6.82)^c^
Track and field (indoor)	3.17 (1.49-6.72)^c^
Track and field (outdoor)	1.73 (0.76-3.92)
Volleyball	2.77 (1.34-5.72)^c^
Water polo	1.65 (0.20-13.81)
Wrestling	1.16 (0.25-5.32)
Other or multiple sports	1.67 (0.65-4.31)
Sport currently in season	1.14 (0.92-1.40)

Abbreviations: NCAA, National Collegiate Athletic Association; OR, odds ratio.

^a^ Risk-adjusted ORs calculated from multivariable logistic regression model (interpersonal violence as outcome).

^b^ Other race and ethnicity included American Indian or Alaskan Native, Asian, Hispanic or Latino, Native Hawaiian or Pacific Islander, non-Hispanic Black, Other, and Multiracial.

^c^ Statistically significant.

### Risk-Adjusted Associations Between IV and Outcomes

Risk-adjusted associations between experiences of IV and outcomes are presented in Table 4. Compared with athletes who did not report experiencing IV, athletes who reported any form of IV were significantly less likely to perceive that they performed well. They were less satisfied with their performance (mean difference on a 10-point Likert scale, −0.68; 95% CI, −0.91 to −0.44; *P* < .001) and felt that they contributed less to their teams’ success (mean difference, −0.67; 95% CI, −0.93 to −0.41; *P* < .001). Such differences were primarily associated with greater extents of psychological or emotional abuse (contributing a minimum of 35.8% to a maximum of 82.5%) and neglect or abandonment (contributing a minimum of 10.4% to a maximum of 39.6%).

**Table 4.** Risk-Adjusted Associations Between Variations in Outcomes and Self-Reported Experiences of Interpersonal Violence (IV)

	Any form of IV		Weighted contribution, %^a^				
	Mean difference (95% CI)^b^	*P* value	Physical	Financial	Sexual	Psychological or emotional	Neglect or abandonment
Perceived performance, assessed with Athlete’s Subjective Performance Scale							
Score range: 1-10, with higher values indicating better performance							
Satisfied with sporting performance	−0.68 (−0.91 to −0.44)	<.001^c^	25.8	2.0	NC	37.6	34.6
Contribution to team success	−0.67 (−0.93 to −0.41)	<.001^c^	NC	18.9	NC	68.1	13.0
Capabilities truly reflected	−0.74 (−0.98 to −0.49)	<.001^c^	11.2	13.7	NC	47.6	27.5
Contribution to improving teammates’ performance	−0.63 (−0.87 to −0.40)	<.001^c^	NC	7.1	NC	82.5	10.4
Functioning during challenging moments	−0.72 (−0.95 to −0.49)	<.001^c^	13.3	11.1	NC	35.8	39.7
Coach satisfaction with performance	−0.35 (−0.45 to −0.25)	<.001^c^	NC	4.2	NC	56.2	39.6
Team cohesion, assessed with Group Environment Questionnaire							
Score range: 1-9, with higher values indicating greater inclusion							
Individual attraction to the group, social	−1.15 (−1.31 to −0.99)	<.001^c^	14.6	18.7	NC	29.6	37.0
Individual attraction to the group, task	−1.54 (−1.73 to −1.36)	<.001^c^	11.5	21.3	5.9	32.8	28.6
Group integration, social	−1.35 (−1.53 to −1.18)	<.001^c^	5.7	16.8	4.7	37.7	35.1
Group integration, task	−1.17 (−1.35 to −1.00)	<.001^c^	4.3	21.0	NC	39.9	34.8
Emotional connection to sport, assessed with SafeSport							
Score range: <2 to ≥2, with higher values indicating less connection							
Feeling burned out by sport participation	0.75 (0.63 to 0.86)	<.001^c^	5.1	13.3	6.1	58.6	16.9
Feeling uninterested in sport participation	0.76 (0.65 to 0.87)	<.001^c^	3.1	12.9	10.7	56.4	16.7
Wishing to be somewhere else during sport	0.83 (0.72 to 0.94)	<.001^c^	3.8	13.0	3.5	61.7	18.1
Considering quitting sport	0.81 (0.70 to 0.92)	<.001^c^	5.5	15.1	14.7	50.4	14.3
Willingness to seek help, assessed with SSOSH scale							
Score range: 1-5, with higher values indicating lower willingness	0.04 (−0.03 to 0.11)	.22	NA	NA	NA	NA	NA

Abbreviations: NA, not applicable; NC, noncontributory; SSOSH, Self-Stigma of Seeking Help.

^a^ Weighted contributions calculated from separate structural equation models.

^b^ Risk-adjusted mean differences calculated from separate multivariable linear regression models (interpersonal violence as explanatory variable).

^c^ Statistically significant.

Differences in team cohesion followed a similar pattern, with reported experiences of IV associated with significantly lower levels of perceived individual attraction to the group (mean difference on a 9-point Likert scale for social activities, −1.15; 95% CI, −1.31 to −0.99; *P* < .001) and an overall lower sense of group integration (mean difference, −1.35; 95% CI, −1.53 to −1.18; *P* < .001) across each of the questionnaire’s 4 domains. Differences in team cohesion were primarily associated with greater extents of psychological or emotional abuse (contributing a minimum of 29.6% to a maximum of 39.9%) and neglect or abandonment (contributing a minimum of 28.6% to a maximum of 37.0%). However, in contrast to associations with perceived performance, reported experiences of physical abuse, financial abuse, and (to a lesser extent) sexual abuse also played an important role, accounting for more than 4.3% of the total variation in each case. Sexual abuse was noncontributory for 2 of the 4 questionnaire domains.

Regarding differences in athletes’ emotional connection to their sport, reported experiences of IV were associated with significantly greater extents of burnout (mean difference on a 5-point Likert scale, 0.75; 95% CI, 0.63-0.86; *P* < .001), lack of interest in continuing (mean difference, 0.76; 95% CI, 0.65-0.87; *P* < .001), wishing to be somewhere else while playing (mean difference, 0.83; 95% CI, 0.72-0.94; *P* < .001), and considering quitting (mean difference, 0.81; 95% CI, 0.70-0.92; *P* < .001). Such associations were primarily associated with greater extents of psychological or emotional abuse (contributing a minimum of 50.4% to a maximum of 61.7%). Neglect or abandonment (contributing a minimum of 14.3% to a maximum of 18.1%) and financial abuse (contributing a minimum of 12.9% to a maximum of 15.1%) each accounted for 10% to 20% of the total variation, whereas reports of physical abuse (contributing a minimum of 3.1% to a maximum of 5.5%) and sexual abuse (contributing a minimum of 3.5% to a maximum of 14.7%) each accounted for approximately 5% to 10% of the total variation. When sexual abuse occurred, it explained up to 14.7% of the total variation in athletes’ desire to quit. Reports of IV were not significantly associated with differences in athletes’ willingness to seek help.

### Risk-Adjusted Associations Between Variations in Coaching Style and IV Reporting 

Variations in coaching styles were associated with differences in IV reporting (Table 5). When looking at differences in perceived support of athlete autonomy, we found that athletes with coaches who were perceived to provide more support for athlete choices; make sure that athletes feel understood; convey confidence in athletes’ ability to do well; and encourage, listen to, or try to understand how athletes see things reported a 3.9 to 5.1 risk-adjusted absolute mean percentage point decrease in the probability of experiencing IV. For example, having access to a coach who made sure that athletes felt understood resulted in a 5.1 (95% CI, 4.5-5.7) risk-adjusted absolute percentage point decrease.

**Table 5.** Risk-Adjusted Associations Between Variations in Coaching Style and Self-Reported Experiences of Interpersonal Violence (IV)

Coaching style	Mean difference (95% CI), percentage points^a^
Athlete autonomy	
Coach provides support with choices and options	−4.2 (−4.6 to −3.5)
Coach makes sure I feel understood	−5.1 (−5.7 to −4.5)
Coach conveys confidence in my ability to do well	−4.6 (−5.4 to −4.0)
Coach encourages me to do well	−4.8 (−5.4 to −4.1)
Coach listens to how I would like to do things	−3.9 (−4.5 to −3.4)
Coach tries to understand how I see things	−4.3 (−4.8 to −3.7)
Overall: athlete autonomy	−4.5 (−5.1 to −3.9)
Team culture	
Coach seeks to understand needs and view of athletes	−6.0 (−7.0 to −5.0)
Coach listens to athletes’ opinions	−6.4 (−7.3 to −5.4)
Coach is devoted to the team	−5.4 (−6.6 to −4.2)
Coach cares about whether we win or lose	−4.3 (−5.5 to −3.1)
Coach accepts responsibility for the impact of their decisions	−6.6 (−7.6 to −5.5)
Coach is committed to helping athletes improve	−6.2 (−7.4 to −5.1)
Coach prioritizes relationships with athletes	−6.1 (−7.1 to −5.1)
Coach reacts thoughtfully to athletes	−7.4 (−8.4 to −6.4)
Coach reacts harshly to athletes	2.0 (1.2 to 2.7)
Coach inspires athletes to purpose beyond winning	−6.1 (−7.2 to −5.1)
Overall: team culture (reversed reacts harshly)	−5.7 (−6.5 to −4.8)
Abusive supervision	
Coach ridicules me	12.0 (10.6 to 13.3)
Coach tells me thoughts or feelings are stupid	11.9 (10.2 to 13.7)
Coach gives me the silent treatment	12.1 (10.7 to 13.6)
Coach puts me down in front of others	12.2 (10.8 to 13.6)
Coach invades my privacy	10.9 (9.0 to 12.9)
Coach reminds me of past mistakes and failures	11.3 (10.0 to 12.6)
Coach does not give me credit for jobs requiring effort	11.3 (10.1 to 12.5)
Coach blames me to save themself embarrassment	13.4 (11.5 to 15.1)
Coach breaks promises they make	10.9 (9.5 to 12.4)
Coach expresses anger at me when they are mad	12.0 (10.7 to 13.4)
Coach makes negative comments about me to others	12.7 (11.1 to 14.2)
Coach is rude to me	15.4 (13.8 to 17.1)
Coach does not allow me to interact with teammates	8.7 (6.5 to 10.6)
Coach tells me I am incompetent	12.0 (10.0 to 13.9)
Coach lies to me	11.5 (9.8 to 13.2)
Overall: abusive supervision	11.9 (9.7 to 13.5)

^a^ Risk-adjusted mean differences calculated from separate multivariable linear regression models (interpersonal violence as outcome). *P* < .001 for all associations.

When looking at differences in team culture, we found that more supportive coaching styles were associated with reduced IV reporting (Table 5). Athletes with coaches who were perceived to react more thoughtfully reported a 7.4 (95% CI, 6.4-8.4) risk-adjusted mean absolute percentage point decrease in the probability of experiencing IV. Similar results were seen for athletes with coaches who sought to understand athletes’ needs (−6.0 [95% CI, −7.0 to −5.0] percentage points), listen to athletes’ opinions (−6.4 [95% CI, −7.3 to −5.4] percentage points), and be devoted to their team (−5.4 [95% CI, −6.6 to −4.2] percentage points). In contrast, athletes with coaches who were perceived to react more harshly reported a 2.0 (95% CI, 1.2-2.7) risk-adjusted percentage point increase in the probability of experiencing IV (95% CI, 1.2-2.7).

When looking at differences in the perceived prevalence of abusive supervision, we found that more abusive coaching was associated with a marked increase in IV reporting (Table 5). Athletes with coaches who were perceived to ridicule players reported a 12.0 (95% CI, 10.6-13.3) risk-adjusted mean absolute percentage point increase in the probability of experiencing IV. Similar results were seen for athletes with coaches perceived as being rude to players (15.4 [95% CI, 13.8-17.1] percentage points), blaming players to save themselves from embarrassment (13.4 [95% CI, 11.5-15.1] percentage points), and making negative comments about players to others (12.7 [95% CI, 11.1-14.2] percentage points).

## Discussion

The results of this national multi-institutional survey demonstrated that among currently competing college athletes, 1 in 10 (404 of 4119) experienced IV in sport during their college career. Athletes who reported IV were most likely to identify as female and have nonheterosexual sexual orientations. While prevalent in all sports, IV was highest among athletes competing in sports involving full contact and predominately female athletes. Athletes who were exposed to IV consistently reported worse outcomes, including increased rates of burnout and an expressed desire to consider quitting their sport. They were less satisfied with their performance and felt less connected to their team but were, encouragingly, not less willing to seek help. Unique to these results was the association found between perceived variations in coaching styles and athlete-reported experiences of IV. On average, we found that having a more supportive coach reduced reports of IV in sport by up to 7.4 percentage points (absolute decrease). In contrast, having a more abusive coach increased reports of IV by up to 15.4 percentage points (absolute increase).

Prior studies of IV in sport have largely focused on the retrospective experiences of former or retired athletes who competed at elite levels outside the US^7,8^ or as children on youth and adolescent athletic teams.^11,12,13,14,15,18^ The result has been a wide range of reported prevalence estimates, a difference that may be at least partially explained by variation in definitions of what constitutes IV and how it is assessed (including the use of screening vs more detailed, scenario-based measures), restriction to IV in sport vs other settings (eg, inclusion of intimate partner violence or childhood abuse), restriction to current vs previous sporting experiences, reliance on retrospective recall, regional and national differences, athletes’ level of participation in competitive sport, and the possibility that IV is poorly understood and/or generally underreported among currently competing athletes. The present study builds on this previous work, providing one of the first comprehensive assessments of self-reported experiences with IV in sport by currently competing college athletes while in the middle of their college career. It expands on emerging research among US college athletes that has, until now, primarily focused on the potentially devastating consequences of sexual abuse as a form of IV.^8,16,17,18,19,20^ Recent research from the Commonwealth Games Federation suggests that athletes who are actively engaged in highly competitive training are in a unique period of their lives.^52^ Qualitative interviews with currently competing adult athletes, who are mentally honed to focus on the all-consuming nature of their sport, suggest that, in the moment that IV occurs, these athletes might not be consciously aware of IV while still forced to cope with IV’s consequences.^52^ Similar patterns have been reported among survivors of other types of trauma and abuse.^53,54,55^ If true, the distinction could help to further explain the lower prevalence of IV identified by currently competing college athletes in this study as well as the consistent association between recognized self-reports of IV and adverse psychosocial and emotional outcomes.

Among the risk factors associated with reports of IV, none had a bigger implication than coaches. A study by Parent and colleagues^17^ conducted among 267 elite collegiate athletes at 6 universities in Switzerland found that, while other students were the most frequent perpetrators of sexual abuse (both within and outside of a sporting context), college athletes were 7 times more likely to identify the influence of a coach. Work by Stirling and Kerr,^56^ conducted as a series of 9 semistructured interviews with retired female athletes, speaks to the “incredible perceived power” (p231) that coaches held over athletes actively competing in high-intensity training. Such examples underscore the critical role of coaches as role models, protectors, and potential perpetrators of IV among college athletes. The results of this study agree, pointing to the direct association between perceived variations in coaching style and reports of IV. The close relationship between athlete and coach in a high-intensity training environment creates an opportunity around which future interventions could be designed, particularly those addressing the needs of student athletes.

Such recommendations echo the growing recognition that to meaningfully address IV in sport on a national and international scale^1,6^ and to reduce disparities that most affect athletes, a multifaceted approach is needed. On an individual and local level, athlete-centered interventions (eg, bystander training)^57,58^ that educate and inform key personnel about the presence and risk factors of IV are important to help reduce initial exposure. Access to support and appropriate mental health care is needed to aid survivors and reduce adverse outcomes. Safe reporting infrastructure (eg, independent disclosure mechanisms and safe contact points)^59^ could further help at the university and regional level. Nationally, organizational and strategic policies need to change^60^ such that the NCAA and other governing bodies begin to acknowledge the ubiquity of IV in college sport, develop accurate prevention and monitoring systems, and ensure appropriate consequences for perpetrators to protect survivors and dissuade further abuse.

Unique among sport settings, the US NCAA is committed to optimally and holistically developing young athletes in its charge. This setting, therefore, has an outsized dependence on healthy and responsible coach-athlete relationships that facilitate mutual dependency, trust, closeness, and care.^3^ The recommendations that these and related data inspire target coaches and their institutions. At the level of the NCAA and individual institutions, healthy coach-athlete relationships can be facilitated by (1) investment in coach education focused on how to use their positions to build trust, mutual respect, and communication^61^; (2) provision of emotional and other support to coaches, with the understanding that it is impossible to “pour from an empty cup” (ie, one needs to feel supported and have sufficient empathetic reserves to provide support and empathy to others)^61^; (3) provision of positive reinforcement and recognition of best-practice coach-athlete relationships that serve as models to others^62^; and (4) promotion of increased accessibility for and responsiveness to athletes in need, recognizing the barriers that athletes need to overcome before reaching out in the first instance.^62^ At the level of individual coaches, the steps to facilitate the development of athlete-centered safe sports environments include (1) acknowledging the ubiquity of IV in sport among team leaders; (2) establishing a diverse and mutually accountable coaching staff who include or can access experts in trauma-informed care; (3) holding space for and listening empathically to athlete feedback; and (4) building a culture of trust, mutual respect, and multiscale communication.^62^

### Limitations

This study has limitations. The most important limitations include its reliance on retrospective, self-reported survey data from a convenience sample of currently competing college athletes who participated in the annual NCAA myPlaybook survey.^40,41^ While believed to be representative of the NCAA athlete population as a whole, given how the survey was designed,^40,41^ the results of this study might differ from the experiences of athletes at institutions that were not included in the sample or athletes who chose not to participate. In looking to study currently competing athletes’ experiences of IV during their college sports career, we intentionally sought brief, subjective reports. Doing so allowed us to garner a deeper understanding of respondents’ lived experience in a nonintrusive manner. However, it should be recognized that despite providing examples and definitions in the survey text, survey responses could vary between respondents; be affected by factors that make respondents more or less likely to remember or identify events; and differ from what actually occurred were the results evaluated using a more objective and/or detailed measure, inclusive of a larger number of more descriptive scenarios or questions.

Due to limited numbers of responses (19 [0.5%]), athletes who did not identify as male or female were excluded from the study. Future research is warranted to explore these athletes’ unique experiences and to reflect the potential for various types of IV to differentially affect specific adverse outcomes that college athletes encounter. Although differences in coaches’ extent of abusive supervision and athlete reports of psychological or emotional abuse and neglect or abandonment measured seemingly similar concepts, analyses of the extent of their overlap revealed differences between the 2 parameters. This finding likely reflects the results reported in previous research that, while coaches working in positions of power are uniquely positioned to exert a protective or provoking role over college athletes, they are not the only (or even the most likely) perpetrators of IV in sport.^17^ Coaches are, however, the people in positions of power with the ability to directly alter the team culture under which other student athletes feel compelled and/or motivated to act.

## Conclusions

Interpersonal violence is associated with marked changes in the psychosocial health and emotional well-being of college athletes. Given the association between IV exposure and reports of adverse outcomes, efforts are needed to reduce IV’s potential harm through both clinical initiatives aimed at secondary screening and tertiary prevention on the part of team physicians, family medicine physicians, emergency department physicians, and primary care physicians.^63,64,65^ Initiatives should also include more direct efforts to promote primary prevention of IV in sport enacted on multiple levels^57,58,59^ that seek to leverage the unique position that coaches and higher-level administrators hold to help reduce IV and create safe places where all college athletes can thrive. As a first step in describing the prevalence, associations with demographic factors, and consequences of experiencing IV in sport among currently competing US college athletes, the results of this study provide a foundation on which future studies are encouraged to build. The results expand definitions of IV from a historical focus on sexual violence to consider how other types of IV (notably including psychological or emotional abuse and neglect or abandonment) disproportionately affect athletes who identify as being female, having a nonheterosexual sexual orientation, or living with a disability.

## Notes

Supplement 1. **eAppendix.** Demographic Variable Definitions, Interpersonal Violence Operational Definitions, and Interpersonal Violence Survey Text

Supplement 2.
Data Sharing Statement
