# Supplementary material for: Experiences of Interpersonal Violence in Sport and Perceived Coaching Style Among College Athletes
Source: JAMA Netw Open. 2024 Jan 16;7(1):e2350248. doi: 10.1001/jamanetworkopen.2023.50248 (PMC10792469; doi:10.1001/jamanetworkopen.2023.50248)
Supplement: Supplement 1. — eAppendix. Demographic Variable Definitions, Interpersonal Violence Operational Definitions, and Interpersonal Violence Survey Text [file jamanetwopen-e2350248-s001.pdf]

Supplemental Online Content

Zogg CK, Runquist III EB, Amick M, et al. Experiences of interpersonal violence in sports and perceived coaching style among college athletes. *JAMA Netw Open*. 2024;7(1):e2350248. doi:10.1001/jamanetworkopen.2023.50248

eAppendix. Demographic Variable Definitions**,** Interpersonal Violence Operational Definitions, and Interpersonal Violence Survey Text

This supplemental material has been provided by the authors to give readers additional information about their work.

© 2024 Zogg CK et al. *JAMA Network Open*

eAppendix. Demographic Variable Definitions, Interpersonal Violence Operational Definitions, and Interpersonal Violence Survey Text

Demographic variable definitions

Athlete/Coach Race/Ethnicity
- Non-Hispanic White
- Non-Hispanic Black
- Hispanic or Latino
- Asian
- Native Hawaiian or Pacific Islander
- Other
- Multiracial

Athlete/Coach Gender Identity
- Male
- Female
- Transgender
- Non-binary
- Other

Athlete Sexual Orientation
- Straight or Heterosexual
- Gay/Lesbian or Homosexual
- Bisexual or Pansexual
- Asexual
- Questioning

Athlete Disability Status
- Documented disability
- No documented disability

Athlete International Student Status
- International student
- Domestic student

Athlete Age
- Continuous (18-25)

Year of NCAA Eligibility
- Continuous (1-5)

NCAA Division
- Division I
- Division II
- Division III

NCAA Sport/Athletic Team
- Baseball
- Basketball

- Beach Volleyball
- Bowling
- Cheer
- Cross Country
- Fencing
- Field Hockey
- Football
- Golf
- Ice Hockey
- Lacrosse
- Rowing
- Skiing
- Soccer
- Softball
- Swimming and Diving
- Tennis
- Track and Field (Indoor)
- Track and Field (Outdoor)
- Volleyball
- Water Polo
- Wrestling
- Other
- Multiple

© 2024 Zogg CK et al. *JAMA Network Open*

Interpersonal violence operational definitions

Physical Abuse:

Being physically harmed through actions such as being pushed, kicked, slapped, or punched

Financial Abuse:

Being financially mistreated or taken advantage of through actions such as funds being inappropriately withheld or used for coercion

Sexual Abuse:

Feeling uncomfortable, afraid, or unsafe due to unwanted sexual advances such as undesired touching, kissing, groping, or fondling

Psychological/Emotional Abuse:

Being bullied, intimidated, threatened, demeaned, humiliated, or shamed

Neglect or Abandonment:

Being inappropriately left alone or left with clear needs unanswered or unmet

© 2024 Zogg CK et al. *JAMA Network Open*

Interpersonal violence survey text

During training, have you ever been or felt physically harmed due to the actions of coaches, teammates, team officials, or team administrators (e.g., unduly pushed, kicked, slapped, punched, or otherwise physically hurt)?

- Over the course of your career
- In the last 6 weeks

During training, have you ever been or felt financially mistreated or taken advantage of by coaches, teammates, team officials, or team administrators (e.g., funds inappropriately withheld, funds used to coerce you, or otherwise financially mistreated)?

- Over the course of your career
- In the last 6 weeks

During training, have you ever been or felt uncomfortable, afraid, or unsafe due to unwanted sexual advances by coaches, teammates, team officials, or team administrators (e.g., undesired touching, kissing, groping, fondling, or otherwise unwanted sexual behavior)?

- Over the course of your career
- In the last 6 weeks

During training, have you ever been or felt psychologically or emotionally mistreated due to the actions of coaches, teammates, team officials, or team administrators (e.g., bullied, intimidated, threatened, demeaned, humiliated, shamed, or otherwise psychologically abused)?

- Over the course of your career
- In the last 6 weeks

During training, have you ever been or felt neglected, isolated, or abandoned due to the actions of coaches, teammates, team officials, or team administrators (e.g., inappropriately left alone, or left with clear needs unanswered/unmet)?

- Over the course of your career
- In the last 6 weeks

© 2024 Zogg CK et al. *JAMA Network Open*
