# Supplementary material for: Experiences of Interpersonal Violence in Sport and Perceived Coaching Style Among College Athletes
Source: JAMA Netw Open. 2024 Jan 16;7(1):e2350248. doi: 10.1001/jamanetworkopen.2023.50248 (PMC10792469; doi:10.1001/jamanetworkopen.2023.50248)
Supplement: Supplement 2. — Data Sharing Statement [file jamanetwopen-e2350248-s002.pdf]

Data Sharing Statement

Data
Data available: No

Additional Information
Explanation for why data not available: Data utilized in the study are available upon request from the University of North Carolina Greensboro Institute to Promote Athlete Health & Wellness. They are not available directly for the study authors due to the inclusion of potentially identifiable respondent information.
